# Looking without Perceiving: Impaired Preattentive Perceptual Grouping in Autism Spectrum Disorder

**DOI:** 10.1371/journal.pone.0158566

**Published:** 2016-06-29

**Authors:** Tiffany A. Carther-Krone, Sarah Shomstein, Jonathan J. Marotta

**Affiliations:** 1 Perception and Action Lab, Department of Psychology, University of Manitoba, Winnipeg, Manitoba, Canada; 2 Department of Psychology, George Washington University, Washington, DC, United States of America; University of Verona, ITALY

## Abstract

Before becoming aware of a visual scene, our perceptual system has organized and selected elements in our environment to which attention should be allocated. Part of this process involves grouping perceptual features into a global whole. Individuals with autism spectrum disorders (ASD) rely on a more local processing strategy, which may be driven by difficulties perceptually grouping stimuli. We tested this notion using a line discrimination task in which two horizontal lines were superimposed on a background of black and white dots organized so that, on occasion, the dots induced the Ponzo illusion if perceptually grouped together. Results showed that even though neither group was aware of the illusion, the ASD group was significantly less likely than typically developing group to make perceptual judgments influenced by the illusion, revealing difficulties in preattentive grouping of visual stimuli. This may explain why individuals with ASD rely on local processing strategies, and offers new insight into the mechanism driving perceptual grouping in the typically developing human brain.

## Introduction

Autism Spectrum Disorder (ASD) is a neurodevelopmental disorder that is associated with three core deficits: abnormalities in communication, poor social behavior, and repetitive or stereotyped behaviors [1]. In addition to the main components classifying an ASD diagnosis, individuals with ASD also have been shown to exhibit superior (faster and more accurate) performance on visuospatial processing tasks in comparison to typically developing individuals. This finding has been supported by studies showing that individuals with ASD show superior performance to typically developing individuals in processing the features of a stimulus compared to stimulus configurations in the block design subtest of the Wechsler Intelligence Scale for Children [2–5], in detecting hidden figures [6] in detection of a local target in a visual search task [7–8], and in feature discrimination [9]. This increased reliance on local details has been explained by two theoretical accounts, the Weak Central Coherence (WCC) theory [10–11] and the Enhanced Perceptual Functioning (EPF) theory [12–13]. The WCC theory suggests that a tendency towards local processing of visual stimuli is a result of a deficit in perceiving a global configuration, while the EPF theory suggests that this tendency is a default visual processing strategy resulting in increased dependence on details.

Although most evidence regarding a tendency towards parts-based processing is found in behavioral studies, some neuroimaging studies have also examined this perceptual abnormality, mainly using the embedded figures task (EFT), which has participants mentally break down complex figures into local parts in order to determine whether one of the parts matches the target figure. Neuroimaging studies have shown that individuals with ASD who are administered this task tend to show less activation in brain regions associated with higher levels of cognitive function and more activation in regions associated with lower levels of cognitive function when compared to controls [14–17]. Ring et al [14] showed that performance on the EFT resulted in greater activation of ventral occipitotemporal regions in individuals with ASD and greater prefrontal activity in typically developing controls, suggesting that individuals with ASD may use different cognitive processing strategies compared to controls when engaging in the EFT. A study by Manjaly et al [15] found brain activation in response to the EFT to be left-lateralized in parietal and premotor areas for typically developing controls, but was found in right primary visual cortex and bilateral extrastriate areas in adolescents with ASD, suggesting that enhanced local processing, rather than impaired processing of global configurations, is characteristic of performance in the EFT by individuals with ASD. Similarly, Damarla et al [16] found that performance on an EFT resulted in greater activation of visuospatial areas in ASD and greater activation of left dorsolateral prefrontal and inferior parietal areas in typically developing controls. Reduced frontal-posterior functional connectivity in ASD was also observed, suggesting that the integration of higher-order brain regions with visuospatial regions may be impaired in individuals with ASD. In line with these findings, decreased medial prefrontal activation and decreased connectivity of this region with posterior regions compared to controls has been found to be associated with a reduction in global-to-local interference in individuals with ASD [17]. Taken together, these studies show that individuals with ASD tend to have increased activation in the occipital and superior parietal regions and reduced activation in the frontal regions during visuospatial tasks such as the EFT, suggesting that visual processing in ASD is more low level and perceptually oriented.

Although behavioral and neuroimaging research has substantially increased the understanding of mechanisms possibly underlying perceptual processing in ASD, the majority of perception studies in ASD have evaluated the notion of a local-processing tendency in conditions where attentional processes are recruited. However, perceptual processing at a preattentive level significantly impacts how information is grouped and integrated into our perception of the world around us. Before attention is even directed to a visual scene, incoming visual information is quickly and efficiently organized so that meaningful perception can occur. The visual system is able to group elements using principles first introduced by Gestalt psychologists, including similarity, proximity and closure [18]. This allows visual input to be organized and integrated from meaningless and fragmented input into coherent, whole objects and backgrounds.

Insights into perceptual processing are often gleaned from exploring visual illusions that rely on perceptual grouping. It has been established that typically developing individuals are susceptible to illusions even at preattentive stages of visual processing [19], suggesting that the ability to group individual features into a coherent whole can occur prior to attentional allocation. Individuals with ASD tend to focus on local features—possibly due to a breakdown of integrating individual features into a whole [2, 20–23]. Research using visual illusions suggests that this local processing bias in ASD is associated with decreased Gestalt perception [24–26], although this has been challenged by research showing lower-level coherence in visual processing is intact [27–28]. While a variety of factors may explain this divergence of evidence, such as type of illusion used, whether a verbal or manual response is required, or instruction, another important factor to consider is the amount of attention directed at the stimuli. Although perceptual grouping is considered by many to be a purely preattentive and automatic process [29–30], this may not be the case for individuals with ASD who may require the recruitment of attentional processes to carry out perceptual grouping tasks. Even though illusion tasks requiring quick, verbal line judgments tend to show decreased Gestalt perception in individuals with ASD [24–26], tasks demanding more attention, such as making manual line adjustments to illusions, tend to indicate that perceptual grouping abilities are intact in these individuals [28,31]. It has been shown that children with ASD use local processing strategies in a divided attention task, but are able to recruit global processing abilities when their attention is directed to it in a selective attention task [32]. However, it is unclear the extent to which attention influences their ability to perceptually organize visual environments.

Here, we present novel findings that challenge the hypothesis that perceptual organization abilities are intact in ASD. By adopting a paradigm originally developed by Moore and Egeth [19], where participants discriminate two lines superimposed on a background designed in such a way as to preattentively (i.e., without conscious awareness) induce the Ponzo illusion, we directly test whether ASD individuals are able to group visual elements preattentively. If individuals with ASD are unable to perceptually organize their environment at early, preattentive stages of visual processing, then their behavior will be inconsistent with the Ponzo illusion. Alternatively, if perceptual grouping abilities are intact at early stages of visual processing, then behavioral performance will be consistent with the Ponzo illusion and will be similar to that of typically developing controls. Additionally, if attention affects the ability to group the background dot pattern, then individuals with ASD should show increased susceptibility to the illusion and increased ability to see and correctly identify the background pattern as it becomes more salient.

To foreshadow our results, we showed that even though neither group was aware of the illusion, individuals with ASD were significantly less likely than typically developing individuals to make perceptual judgments influenced by the illusion. At the end of the experiment participants were also questioned on their ability to identify the background pattern under preattentive, divided attention, and full attention conditions. Although both groups showed increased susceptibility to the illusion on the divided attention trials compared to the preattentive trials, this difference was not statistically significant. However, the ability to see and correctly identify the pattern increased as more attentional processes were directed to the background stimuli, indicating that the dots forming the converging lines can be perceived when attention is explicitly directed to them.

## Methods

### Participants

Two groups were recruited: a high-functioning ASD group included 15 adults (10 males; 1 left-handed; age range 18–70 years, *M* = 35 years) and a typically developing (TD) group consisted of 15 individuals matched on the basis of sex, handedness and chronological age (10 males; 1 left-handed; age range 18–69 years, *M* = 35 years). All individuals with ASD were formally diagnosed with Asperger’s. Verbal and non-verbal cognitive abilities were measured in the ASD group using the Raven’s Standard Progressive Matrices (RSPM; a measure of nonverbal mental ability) [33] and the Peabody Picture Vocabulary Test (PPVT; a measure of verbal ability) [34]. The individuals with ASD had RSPM (*mean* = 44.87, *SD* = 7.47) and PPVT (*mean standard* = 109.4, *SD* = 11.36) scores that were consistent with previous research involving both adults with high-functioning ASD and typically developing adults [35–38], indicating that both verbal and non-verbal cognitive abilities were similar to those of typically developing individuals. Typically developing individuals had all completed high school and were assumed to have a mental age to be similar to their chronological age.

All participants had normal or corrected-to-normal vision. The number of participants was preset to 15–20 based on comparable recent research involving visual illusions in both individuals with ASD and TD individuals [19, 24, 39]. All participants provided written consent, and this study was approved by the Psychology/Sociology Human Research Ethics Board (P2013:034) at the University of Manitoba.

### Stimuli

**Fig 1:**
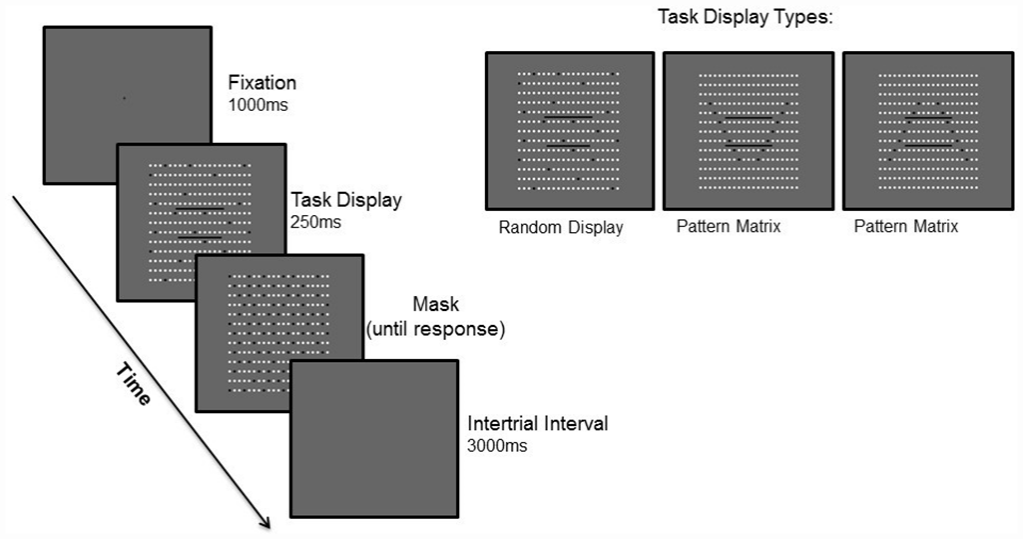
Stimulus Backgrounds and Time Course of Trial Events.

Random matrix trials consisted of two horizontal line segments superimposed on a background of randomly generated black and white dots, where one line segment was physically longer than the other (Fig 1). Unlike the random matrix trials in which the horizontal lines physically differed from one another, pattern matrix trials consisted of two equal horizontal line segments. Importantly, the background of black and white dots, within which the line segments were presented, was ordered such that the black dots would generate two converging lines if the dots were grouped together (Fig 1). This pattern arrangement induces the Ponzo illusion (aka the railroad tracks illusion) such that the two identical horizontal lines appear to be of different lengths. Half of these trials showed the illusion with the two lines converging at the bottom while the other half showed the illusion with the two lines converging at the top. The two equal line segments used for discrimination were centered within this pattern of converging lines, which was centered within the dotted matrix.

### Procedure

Participants were tested either in their homes or at the University of Manitoba’s *Perception and Action Lab*. The experiments were run using a script constructed in E-Prime [40]. Stimuli were presented on a 17” Apple MacBook Pro-laptop.

Before the experiment began, participants were verbally given the instructions for the experiment. Written instructions were also displayed on the computer screen before each subsection of the experiment (practice trials, illusion trials and attention trials). During the experiment, participants were asked to fixate on a central cross that was presented for 1000ms at the start of each trial (Fig 1). This was followed by the trial display for 250ms, after which a mask display appeared. The mask remained on the screen until the participant made a keypress response, and an intertrial interval of 3000ms then followed to indicate the end of the trial.

**Fig 2:**
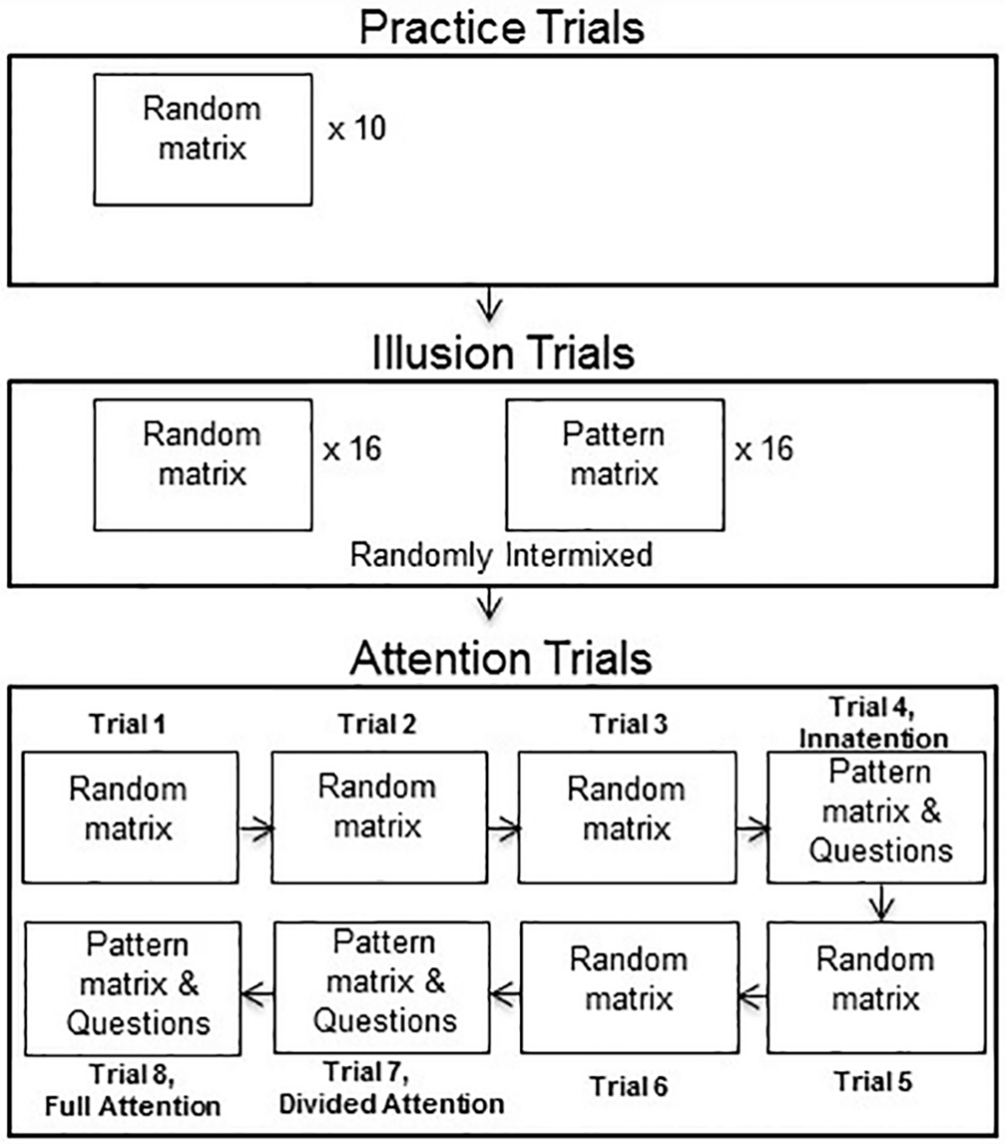
Schematic diagram showing the experimental structure.

The experiment began with a set of 10 practice random matrix trials, allowing participants to adjust to the quick presentation of the stimuli (Fig 2). Following practice, participants completed 32 trials—16 random and 16 pattern matrix trials, randomly intermixed. Finally, a set of 8 probe trials were administered to assess performance on line judgements with various conditions of attention. Of these 8 trials, the first three, as well as the fifth and sixth trials appeared as random matrix trials, while the fourth, seventh, and eighth trials appeared as pattern matrix trials (Fig 2). All trials required participants to indicate which line was longer except for the eighth trial, which did not require a response. Responses were made manually on a keyboard, with participants pressing ‘1’ to indicate the top line was longer and ‘2’ to indicate the bottom line was longer. During the eighth trial participants were asked to ignore the line segment and look for the pattern in the matrix. Immediately following these three pattern matrix trials, three questions were asked to assess the degree of explicit awareness regarding the presence of the background matrix. The first question asked participants to indicate whether or not they had noticed a pattern in the background of the dots on the preceding trial, pressing ‘1’ on the keyboard for *yes* and ‘2’ for *no*. The second question examined whether participants were aware that the background was organized into a particular pattern—participants were shown the two patterns and asked to indicate which pattern matched their observation, pressing ‘1’ on the keyboard for the image presented on the left side of the screen and ‘2’ for the image presented on the right side of the screen. If they did not see a pattern, participants were instructed to press ‘5’ on the keyboard. Finally, participants were asked to provide a confidence rating of how confident they were in their response to the pattern by pressing ‘1’, ‘2’ or ‘3’ on they keyboard, where ‘1’ meant *not at all*, and ‘3’ meant *very confident*. These questions were asked in order to assess whether participants were aware of the background pattern, if they were able to accurately determine the background pattern, and how confident they were that they had seen the background pattern.

Since the background matrix was task-irrelevant and since participants were not told they would be asked questions about the background pattern before they were presented with the first pattern matrix in the set of 8 probe trials (Trial 4), this trial served as an inattention trial. The second pattern matrix trial (Trial 7) was a divided-attention trial since participants may have been prepared for another set of questions to be asked, and as such may have been distributing their attention to both the background task display and the horizontal line segments. The final pattern matrix trial in this set (Trial 8) was defined as a full-attention trial because participants were directed to only pay attention to the background pattern. These probe trials served the purpose of 1) ensuring the stimuli were being viewed under conditions in which the participant was not aware of the pattern in the background, and 2) allowing for observation of how various conditions of attention may affect perceptual grouping abilities. Completion of the attention trials marked the end of the experiment.

## Results

### Illusion Trials

**Fig 3:**
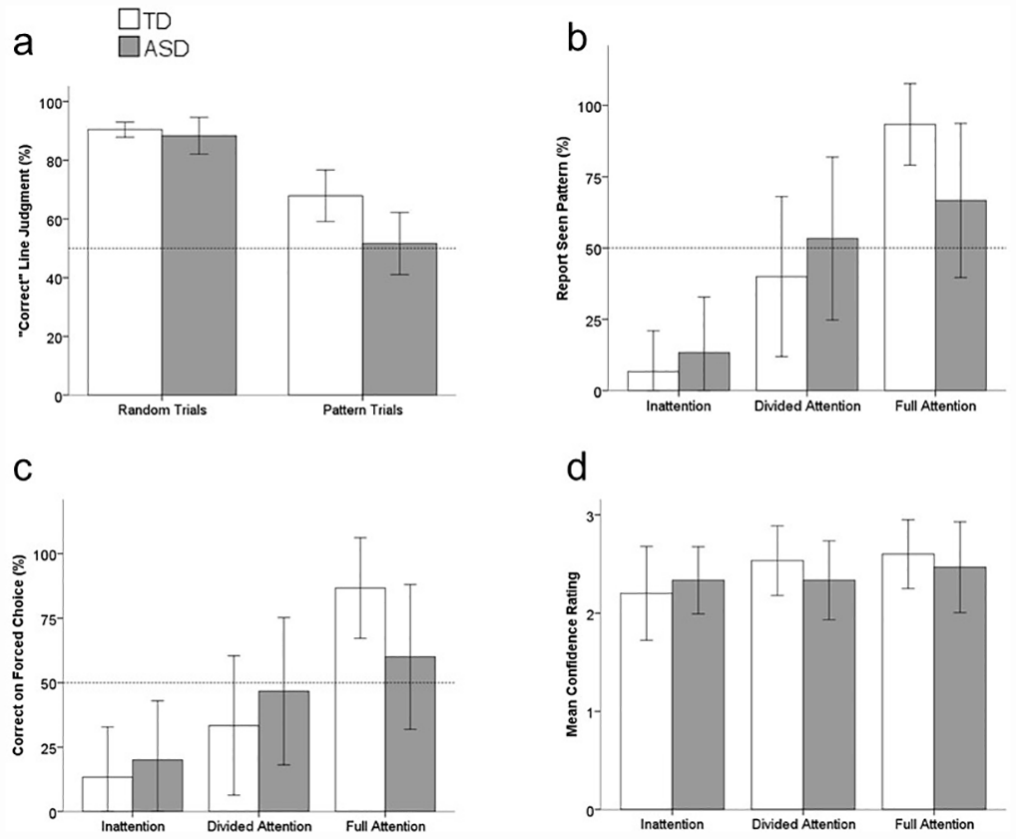
Results of the Perceptual Grouping Task. Figures show the percentage of “correct" line judgments made in the illusion trials (a), the percentage of participants who reported having seen a pattern in response to the direct query (b), the percentage of participants who answered the forced-choice question correctly (c), and the mean confidence ratings of the response given to the forced-choice question (d). Error bars represent 95% confidence intervals.

In the first part of the experiment, preattentive perceptual grouping abilities in individuals with ASD were examined by assessing their susceptibility to the Ponzo illusion in the perceptual grouping task. Fig 3A shows the pattern of performance observed for both pattern and random matrix trials in the illusion set. A “correct” line judgment was coded when participants chose the longer line in the random matrix trials (i.e., the line that was physically longer) or indicated an illusion-based response in the pattern matrix trials (i.e., chose a physically identical line that was consistent with the illusory percept). Perceptual grouping abilities were assessed in both individuals with ASD and typically developing individuals by comparing illusion susceptibility to a chance level (50%) for both pattern- and random-matrix conditions.

The random matrix trials served to ensure that the participants could in fact make perceptual judgments based on line lengths. Both groups showed high accuracy in identifying the longer line. Individuals in the TD group correctly identified the longer line segment on 90.42% (± 2.35%) of the trials, which differed significantly from a 50% chance result, *t*(14) = 33.698, *p* < .001, Cohen’s *d* = 8.69 (Values of the form x ± y refer to a 95% confidence interval of y, surrounding a mean of x). Similarly, individuals in the ASD group correctly reported the longer line segment on 88.33% (± 5.72%) of trials, which also differed significantly from 50%, *t*(14) = 13.143, *p* < .001, Cohen’s *d* = 3.39. An independent groups t-test revealed that there were no significant differences between the two groups, *t*(28) = -.661, *p* > .250, Cohen’s *d* = .25.

**Fig 4:**
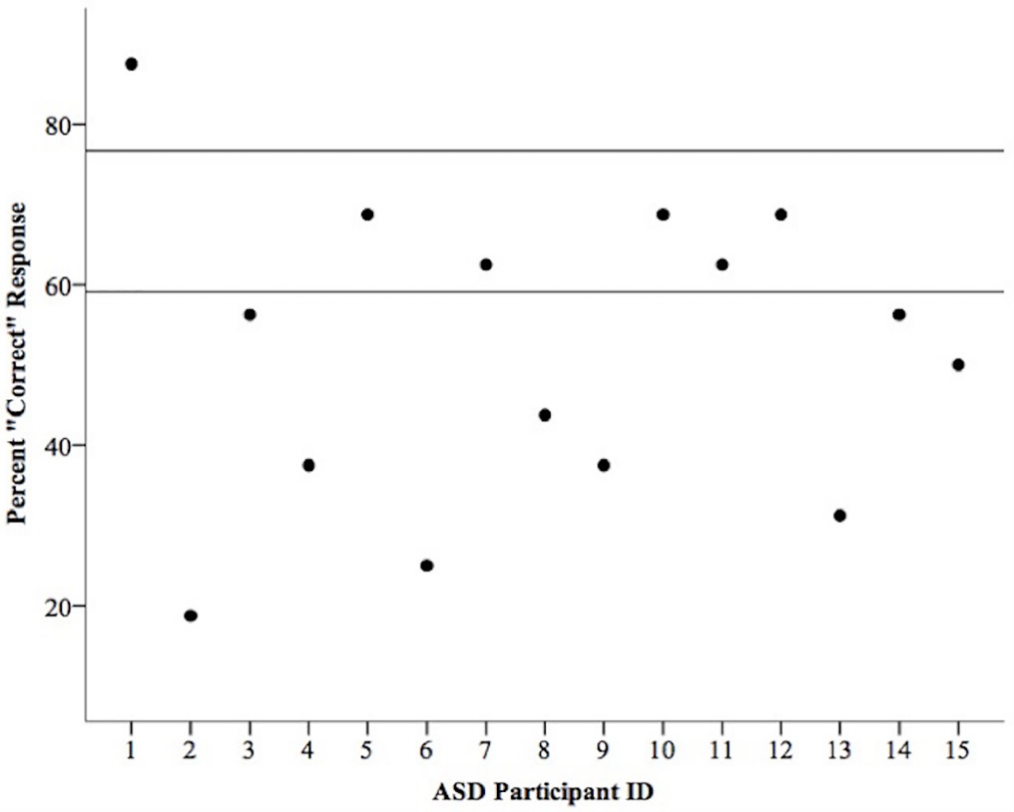
Individual results of the Perceptual Grouping Task for the ASD group. Lines represent the 95% confidence interval of the typically developing group.

The results from the pattern matrix trials were consistent with previous research [19], showing that individuals in the TD group were influenced by the pattern in the background, reporting the line toward the converging end of the patterned lines on 67.92% (± 8.01%) of the trials, which differed significantly from a 50% chance result, *t*(14) = 4.385, *p* = .001, Cohen’s *d* = 1.13. Importantly, individuals with ASD exhibited behavior consistent with the prediction that perceptual grouping does not occur preattentively. Namely, individuals in the ASD group reported an illusion based response on only 51.67% (± 9.62%) of the trials, which did not differ significantly from 50%, *t*(14) = .338, *p* > .250, Cohen’s *d* = .09. An independent groups t-test revealed that the two groups differed significantly, *t*(28) = -2.536, *p* = .017, Cohen’s *d* = .93. To determine the extent to which this finding is consistent at an individual level, the individual data from the ASD group were compared to the 95% confidence interval of the TD group. This revealed that only 5/15 individuals with ASD fell within the 95% confidence interval of the TD group, showing that our findings are supported by responses in all but a small subset of our sample size (Fig 4). These results strongly suggest that individuals with ASD have difficulties perceptually grouping stimuli at early, preattentive stages of visual processing.

### Attention Trials

In the second part of the experiment, we probed whether resistance to visual illusions in ASD individuals was due to a conscious awareness of illusion-inducing elements. In the next analysis, we demonstrate that individuals with ASD, even when consciously aware of the organization of the background, are still unable to perceptually group stimuli in order to perceive the illusion.

As in the first part of the experiment, participants in both groups were highly accurate when asked to identify the longer line on the random matrix trials (93.33% (± 6.25%) for the ASD group and 96.0% (± 4.19%) for the TD group). In the pattern matrix trials presented in the fourth, seventh and eighth trials, participants were guided to increase their awareness of the background pattern. As such, these pattern matrix trials were the inattention, divided-attention, and full-attention trials, respectively. On the inattention trial (Trial 4), the percentage of participants who reported an illusion based response was 46.67% (± 26.13%) for the TD group and 53.33% (± 26.13%) for the ASD group, both of which were not reliably greater than a 50% chance finding (TD: *t*(14) = -.250, *p* > .250; Cohen’s *d* = .064; ASD: *t*(14) = .250, *p* > .250; Cohen’s *d* = .064). On the divided attention trial (Trial 7), 80% (± 20.95%) of individuals in the TD group reported an illusion based response, which was reliably greater than 50%, *t*(14) = 2.806, *p* = .014; Cohen’s *d* = .725. However, only 66.67% (± 24.69%) of individuals in the ASD group reported an illusion based response, which was not reliably greater than the 50% expected due to chance, *t*(14) = 1.323, *p* = .207; Cohen’s *d* = .342. No response was made following the full-attention trial (Trial 8) as participants were asked to only focus on the background dot matrix. These results revealed that regardless of whether the stimuli were presented under conditions of inattention or divided attention, individuals with ASD were not influence by the background dot patterns forming the Ponzo illusion.

To assess whether or not participants were making their line judgments based on an ability to see the background pattern, participants were asked three follow-up questions (the direct query, the forced choice, and the confidence rating) after each of the pattern matrix trials. Fig 3B–3D shows the pattern of performance observed for each of these questions. For each group, a chi-square analysis was performed to examine if there were any relationships of each of the three questions (direct query, forced choice, confidence rating) with attentional type (Inattention, Divided-Attention, Full-Attention). A significant association between results of the direct query question and attention type (Fig 3B) was found for both ASD (*χ*^*2*^(2, N = 45) = 9.36, *p* = .009) and TD (*χ*^*2*^(2, N = 45) = 23.036, *p* < .001) groups. The results of the direct question demonstrate that for both groups, as attention to the background increases (with each subsequent question), the ability to see the background pattern also increases. A significant association between the results of the forced choice question and attention type (Fig 3C) was also found for the TD group (*χ*^*2*^(2, N = 45) = 17.46, *p* < .001), but there was insufficient evidence to show an association between attention type and the forced choice question for the ASD group (*χ*^*2*^(2, N = 45) = 5.101, *p* = .078). The results of the forced choice question suggest that for the TD group, as attentional type increases, so does the ability to correctly identify the background pattern. While individuals with ASD also show this same trend, the changes were not as strong as seen in the TD group. Mean confidence ratings remained relatively consistent throughout all these trials (Fig 3D) and as a result, no relationship was found between the confidence ratings and attention type for neither the ASD group (*χ*^*2*^(4, N = 45) = 5.63, *p* = .228) or TD group (*χ*^*2*^(4, N = 45) = 3.692, *p* = .449).

### Correlation between forced choice and experience of the illusion

To ensure that the illusion was observed at preattentive levels of processing, a point-biserial correlation was calculated between the accuracy of the forced-choice response following the inattention trial and the average percentage of the 16 pattern matrix trials in the set of illusion trials on which an illusion based response was reported. A significant, positive correlation would suggest that reports indicating an illusion-based response may have been the result of participants noticing the patterns on the pattern matrix trials. However, no relationship was found between accuracy of the forced-choice response following the inattention trial and the percentage of pattern matrix trials in the illusion block on which an illusion based response was reported for individuals in the TD group, *r*(15) = -.380, *p* = .163, or for individuals in the ASD group, *r*(15) = .293, *p* > .250. Thus, it is likely that those individuals who fell susceptible to the illusion did so at a preattentive level.

## Discussion

Our results show that individuals with ASD are not susceptible to the Ponzo illusion at the earliest stages of visual processing—before attentional allocation or explicit awareness of the illusion. Individuals with ASD seem unable to group the black background dots, by similarity, into pattern lines. This finding is suggestive of two striking points: 1) that perceptual grouping is not a fully preattentive, automatic process in individuals with ASD; and 2) that varying degrees of attention influences perceptual grouping abilities in individuals with ASD.

Although both groups were poor at indicating which pattern they saw after the inattention trial, the significantly larger amount of illusion-based responses in the typically developing group suggests that despite being unable to explicitly report the pattern, these individuals were still able to implicitly process the pattern, similar to the findings by Moore and Egeth [19]. Individuals with ASD did not show this same ability, suggesting difficulties in preattentive perceptual grouping. Decreased susceptibility to the visual illusion suggests that individuals with ASD were focused more on the individual parts of the stimuli as opposed to the global configuration. This finding not only supports the notion of a local visual processing bias in ASD [3–4, 24–26] but also extends these findings to include perceptual processing at a preattentive level.

Similar results were also seen in the divided-attention condition. Although there was an increase in the number of individuals who were able to accurately see and identify the pattern, about half of the participants in each group were still unable to correctly identify which pattern they saw after the divided-attention trial. However, the typically developing group continued to show a significantly larger number of illusion-based responses, suggesting that even those who were unable to report the pattern were still implicitly processing it. The number of individuals with ASD who showed an illusion based response did not differ from a chance finding, suggesting that they were not aware of the background pattern and that they were still unable to perceptually group the stimuli to perceive the illusion. When attention was directed specifically to the background pattern, individuals with ASD were able to easily identify the pattern, as indicated by a significant increase in the number of individuals who correctly identified the pattern in the full-attention trial compared to the divided-attention trial, indicating that individuals with ASD are able to group the stimuli together when their full attention is directed at the stimuli. These results extend those results of Plaisted and colleagues [32], showing that adults also favor local processing in a divided-attention task, but can recruit global processing abilities to group the stimuli into a whole when attention is specifically directed to it.

One of the critical findings of this research is that individuals with ASD are not able to group the stimuli under preattentive conditions. Yet, other researchers have shown that these individuals do fall susceptible to illusions, particularly under selective-attention conditions or under conditions where more attention is allocated towards the stimuli [28, 32]. These differences in the ability to carry out local and global processing tasks are important to consider in examining the underlying mechanism driving perceptual grouping in the typically developing human brain. In typically developing individuals, global processing strategies are generally recruited regardless of the amount of attention (or lack thereof) directed at the stimuli, unless perception is manipulated so that it is directed at local features, suggesting that the underlying neural mechanism for global processing is similar under all attentional conditions. Further support for this comes from patients with simultagnosia, who present with lesions to the posterior parietal cortex and are not able to process global configurations under any attentional or preattentive conditions [41–42], suggesting that global processing is an “all-or-none” type phenomenon reliant on an intact posterior parietal cortex. However, since we have shown here that individuals with ASD have difficulties in global processing under preattentive conditions, whereas prior research has shown intact global processing when more attention is directed at the stimuli [27–28, 32], this suggests that local and global processing may be occurring on a spectrum, possibly mediated by the recruitment of attentional processes. As such, the ASD brain may serve as a useful model for further examining the underlying neural structures responsible for global processing in the typically developing brain.

An alternative interpretation of this finding is that the lack of susceptibility to the illusion in the ASD group is due to insufficient attentional selection. Since perception is often mediated by attention, it is possible that individuals with ASD have a specific disorder in automatic orienting and zooming out their attentional focus. This attention orienting is comparable to a “spotlight” moving around in visual space, resulting in improved information processing in the attended area at the expense of other non-attended locations [43, 44]. This “spotlight” can not only be directed to specific locations in visual space, but can also be adjusted in size, allowing visual stimuli to be processed either narrowly (zoom-in) or broadly (zoom-out). Previous research has shown that a perceptual integration deficit in children with ASD may be partially due to a zoom-out attentional dysfunction, and that this disorder is specifically linked to the grouping skills involved in coherent dot motion perception [45, 46]. This attentional zoom-out deficit has also been shown to correlate with the severity of social and communicative disorders in individuals with ASD [46]. Thus, the lack of susceptibility to the illusion in our ASD group may have been a result of difficulties “zooming-out” attentional focus, causing these individuals to focus solely on the two horizontal line stimuli at the expense of the rest of the background stimuli. Even though this alternative interpretation is compatible with a local bias in processing, as well as the attentional disengagement disorder in ASD, our inclination towards a preattentive processing deficit comes from the finding that neither the ASD nor the typically developing group indicated seeing the background pattern when probed about it on the inattention trial, suggesting that attention was not explicitly allocated to the background dots in either group.

Although our data support the finding that as a group, individuals with ASD are less susceptible to the Ponzo illusion, it is important to note that a small subset of the ASD group was able to carry out the task in a similar manner to the TD population. This not only demonstrates the heterogeneity that lies within the ASD population, but offers an alternative explanation for discrepancies found within the illusion research, suggesting that differences within the ASD population itself may be influencing the results of local and global processing tasks, a matter of which requires further investigation.

## Conclusions

Taken together, the results of this study provide new insights regarding the mechanism by which individuals with ASD perceptually organize incoming visual information constituting strong support for a local processing bias. Here we have shown that individuals with ASD have difficulties preattentively grouping stimuli into a coherent whole. Our divided- and full-attention trials suggest that varying degrees of attention may influence perceptual grouping abilities, however further research should investigate the extent that this is the case. An important impact of these findings is the effect it may have on social abilities in individuals with ASD. Social stimuli are often made up of complex elements that require integration into a global whole in order to be properly perceived, such as in face processing [47]. As social impairment is one of the three defining characteristics of ASD, understanding the underlying mechanisms of preattentive perceptual grouping may help explain fundamental causes of social impairment in ASD. Future investigations examining brain regions activated during tasks requiring preattentive perceptual grouping in both groups will further our understanding of the processes involved in perceptual organization in ASD.
